# Simultaneous operations for gastric cancer and aortic aneurysm: a case report

**DOI:** 10.1186/s13256-023-03843-y

**Published:** 2023-05-31

**Authors:** Roman Komarov, Sergey Osminin, Stanislav Chernyavsky, Ivan Ivashov

**Affiliations:** 1 Department of Faculty Surgery No 1, Federal State Autonomous Educational Institution of Higher Education I.M. Sechenov First Moscow State Medical University of the Ministry of Health of the Russian Federation (Sechenov University), Bolshaya Pirogovskaya Street 6, Moscow, 119435 Russia; 2 Department of Faculty Surgery No 2, Federal State Autonomous Educational Institution of Higher Education I.M. Sechenov First Moscow State Medical University of the Ministry of Health of the Russian Federation (Sechenov University), Dovator Street 15, Moscow, 119048 Russia

**Keywords:** Gastric cancer, Aneurysm of the abdominal aorta, Surgery

## Abstract

**Background:**

Gastric cancer is the second highest cause of morbidity among malignant tumors of the gastrointestinal tract and fifth in overall cancer statistics. Diseases of the cardiovascular system are the leading causes of death in the world. Aneurysm of the abdominal aorta is the most common type of vascular aneurysm, while in 75% of the cases it is asymptomatic. The risk of rupture of aneurysm of the abdominal aorta increases progressively depending on its diameter and the age of the patient.

**Case presentation:**

A 56-year-old male patient underwent treatment for complaints of pain and discomfort in the epigastric region, general weakness and difficulty in passing food through the esophagus. The neoplasm extended to the esophagus up to 17–20 mm (pT3N3aM0 R0 stage IIIB TNM 8). The aortic diameter at the level of the renal arteries was 18 mm; lower than the main renal arteries, an expansion of up to 60 mm was visualized; the length of aneurysm was 105 mm extending to the bifurcation. A gastrectomy with a resection of the lower thoracic esophagus and application of a manual double-row Roux-en -Y esophagojejunal anastomosis with cholecystectomy and D2 lymphadenectomy was done along with longitudinal aneurysmectomy and thrombectomy. The proximal anastomosis between the aorta and the synthetic linear prosthesis of 18 × 9 × 9 mm in the end-to-end type was formed by a continuous winding suture with the “Prolene” 5-0 thread. The end-to-end distal anastomosis of the prosthesis and aorta branch was formed by continuous winding suture with the “Prolene” 6-0 thread. The postoperative period proceeded without features and complications. On the 7th day after the surgery, the patient was discharged home in satisfactory condition.

**Conclusions:**

Performing a simultaneous operation allowed the patient to undergo rehabilitation after the treatment of two diseases during one hospitalization and, in the shortest possible time, to proceed to the next stage of gastric cancer treatment—chemotherapy, thereby improving the prognosis of life expectancy. Also, one-stage surgical treatment of concomitant aneurysm of the abdominal aorta and gastric cancer is well tolerated and can avoid financial costs, and patient anxiety involved in a second operation.

## Background

Gastric cancer (GC) is the second highest cause of morbidity among malignant tumors of the gastrointestinal tract and fifth in overall cancer statistics. More than 783,000 people die of stomach cancer worldwide each year [1]. Diseases of the cardiovascular system (CVS) are the leading causes of death in the population of developed and developing countries. Thus, according to the World Health Organization (WHO), in 2016, 17.9 million people died from cardiovascular diseases (CVD) in the world, which accounted for 31% of all deaths, and CVD caused disability in 14.8% [1, 2]. Aneurysm of the abdominal aorta (AAA) is the most common type of vascular aneurysm, while in 75% of the cases it is asymptomatic. The risk of rupture of AAA increases progressively depending on its diameter and the age of the patient.

## Case presentation

**Fig. 1:**
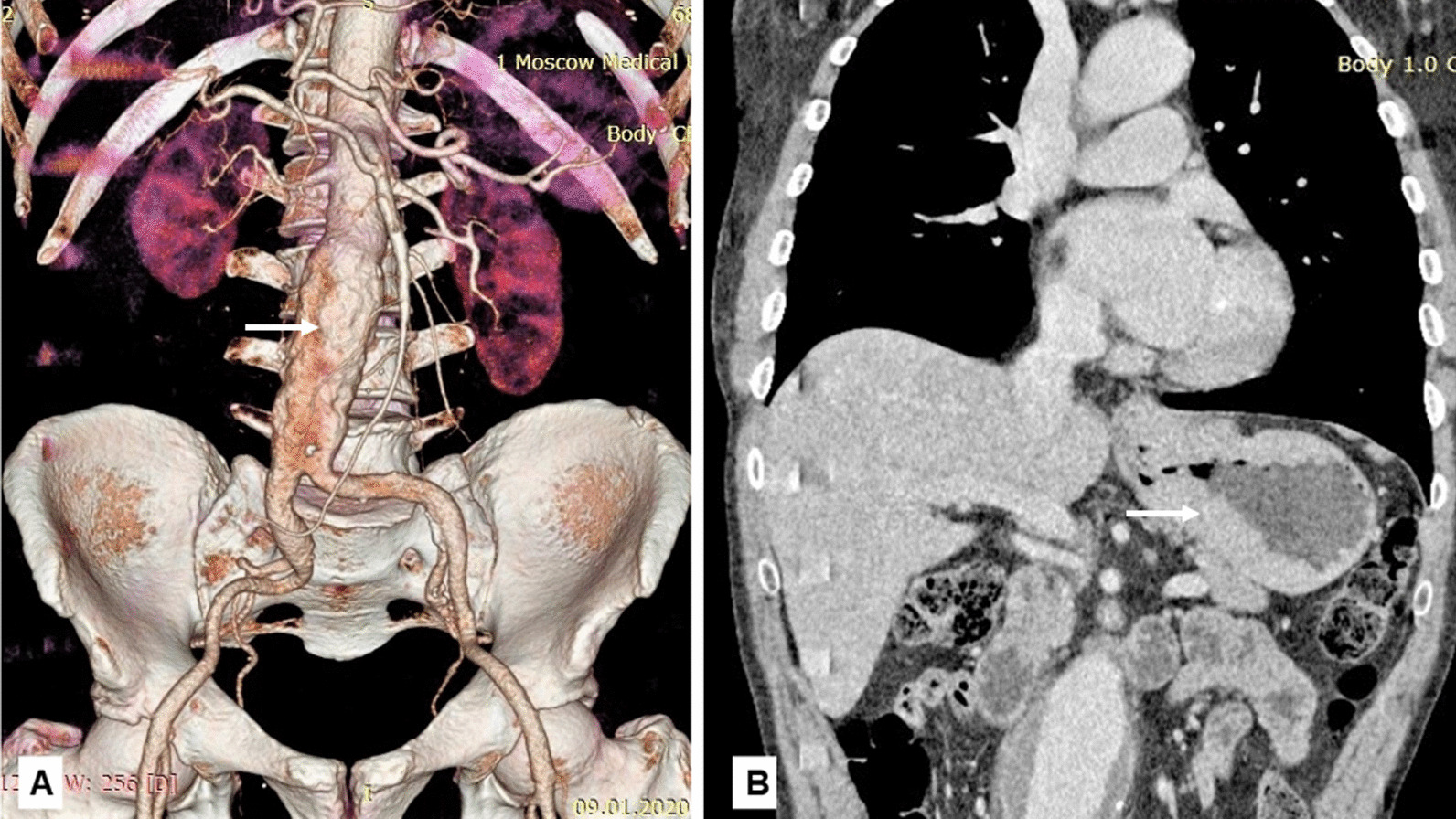
Computed tomography scans of the patient before surgery: **A** 3D reconstruction (the arrow indicates abdominal aortic aneurysm); **B** computed tomography scan of the chest and abdomen (the arrow indicates the lesion of the stomach)

**Fig. 2:**
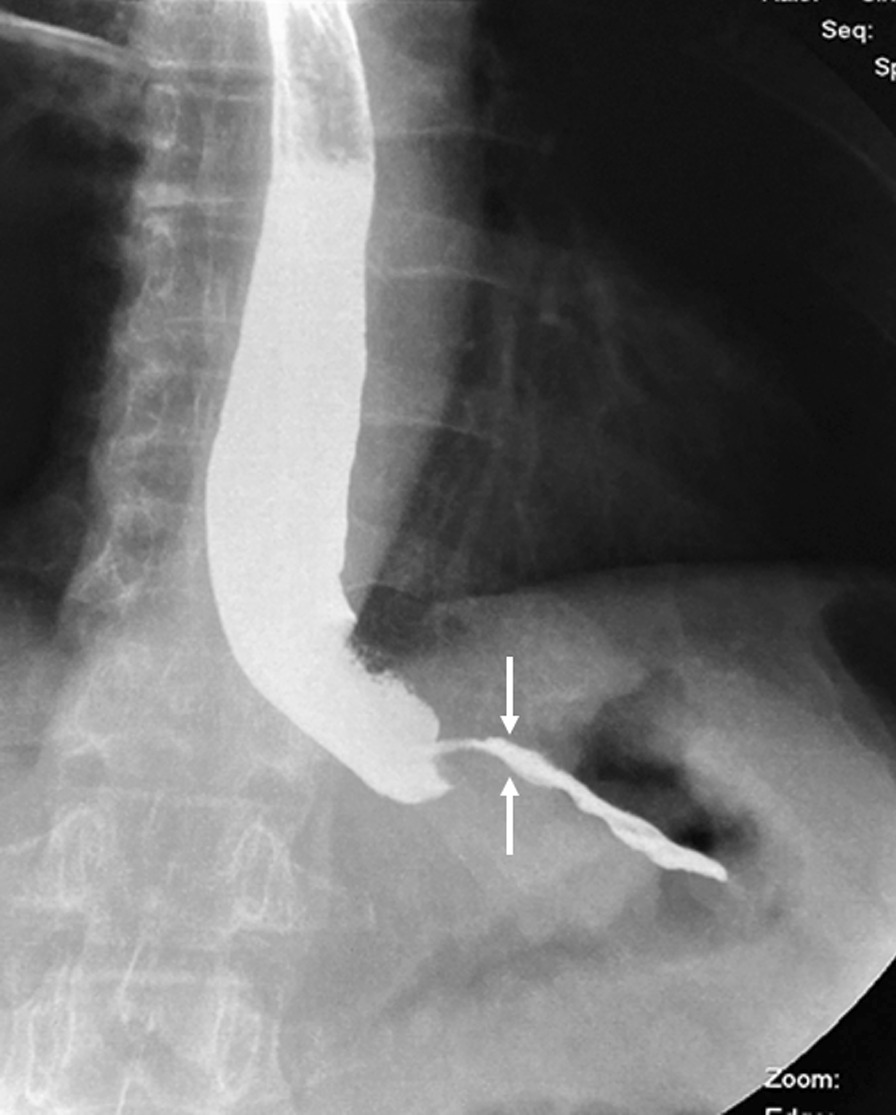
Preoperative patient’s x-ray (the arrows show the narrowed lumen of the stomach by the tumor)

A 56-year-old male patient underwent treatment at the clinic of faculty surgery of Sechenov University for complaints of pain and discomfort in the epigastric region, general weakness and difficulty in passing food through the esophagus. The physical status of the patient was consistent with ASA II. On esophagogastroduodenoscopy: the mucous membrane of the esophagus was gray. The mucous membrane of the stomach was pale pink. In the cardiac region, a voluminous neoplasm was detected, dense, bleeding during a biopsy and spreading to the cardiac part of the esophagus. The neoplasm made it difficult to insert an endoscope into the stomach. The histological examination allowed to diagnose a low-grade adenocarcinoma of the stomach. On computed tomography (CT) (Fig. 1) and x-ray examination (Fig. 2) of the abdominal organs with intravenous contrast: the wall of the stomach in the proximal region, along the lesser curvature over a large area, was unevenly thickened to 27 mm; actively accumulating the contrast drug. The outer contour of the wall in this area was clear, paragastric lymph nodes were detected with a size of 14 mm (in a large number and size along the lesser curvature). The neoplasm extended to the esophagus up to 17–20 mm (invasion?). The aortic diameter at the level of the renal arteries was 18 mm; in the infrarenal region, 20 mm lower than the main renal arteries. An expansion of up to 60 mm was visualized, with parietal thrombosis particularly pronounced along the anterior wall, 20 mm thick; a thrombus with diffusely uneven calcification. The length of aneurysm was 105 mm extended to the bifurcation.

**Fig. 3:**
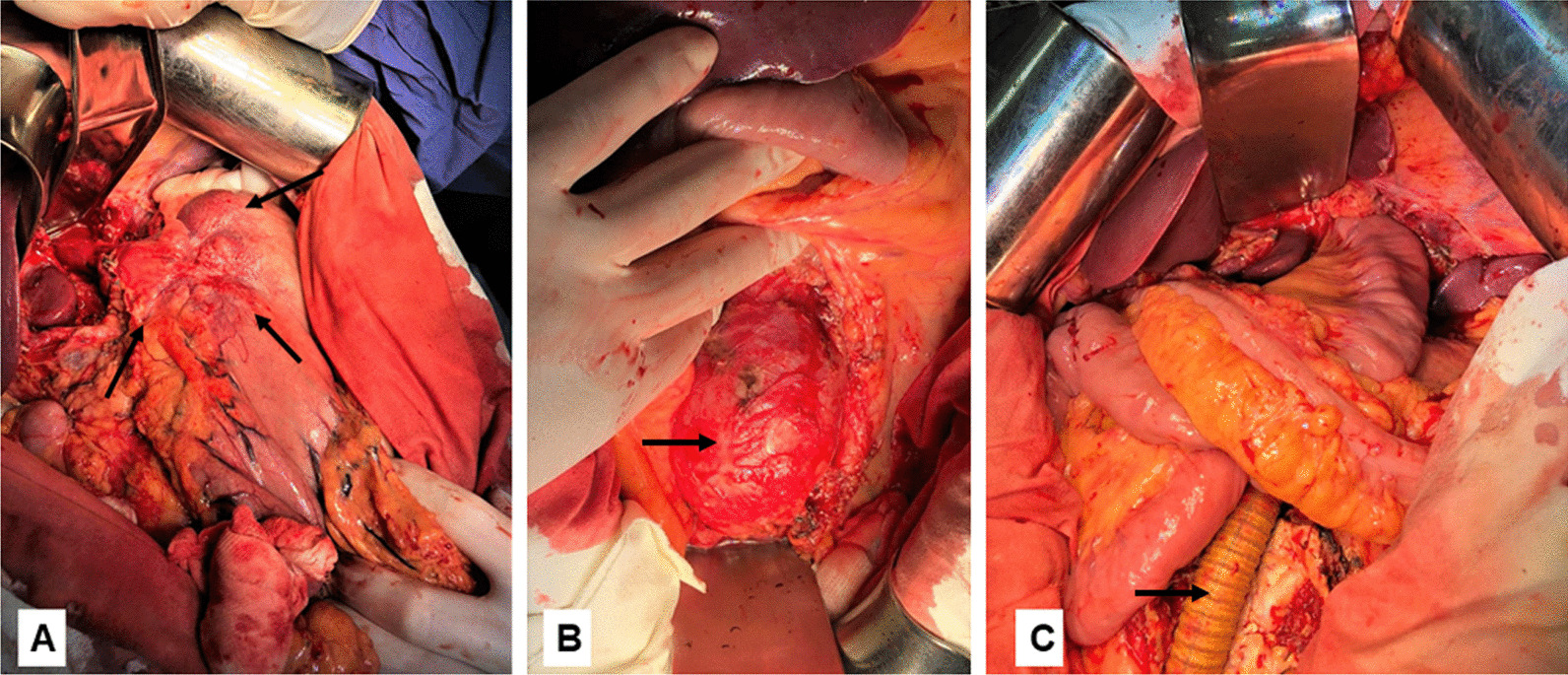
Intraoperative view: **A** stomach cancer (indicated by arrows); **B** abdominal aorta aneurysm (indicated by an arrow); **C** final intraoperative view after reconstruction (the arrow indicates the prosthesis of the abdominal aorta)

The patient underwent laparotomy. During the check-up of the abdominal cavity, no ascites, distant metastases were detected. The liver was macroscopically unchanged. Regional lymph nodes enlarged to 12–16 mm, were identified along the vessels of the celiac trunk paracardiacally. In the cardiac region of the stomach spreading to the bottom and to the middle third of the stomach body, mainly along the lesser curvature with transition to the anterior and posterior walls and spreading to the abdominal esophagus, a tumor of 10 × 5 cm in size was identified (Fig. 3A), sprouting the serous cover, growing into the left crus of the diaphragm.

No changes in the large and small intestine were detected. The gall bladder was not enlarged, its walls were unchanged, it did not contain concrements. In the infrarenal section of the aorta, aneurysmal expansion of up to 10 cm long was detected, aortic diameter up to 65 mm, not extending to the iliac vessels. A gastrectomy was performed with a resection of the abdominal and lower thoracic esophagus with the application of a manual double-row Roux-en -Y esophagojejunal anastomosis, as well as cholecystectomy and D2 lymphadenectomy. Abdominal cavity was lavaged with 2 L of antiseptic solutions. The parietal peritoneum was dissected. The infra-renal section of the aorta was up to 7 cm in diameter (Fig. 3B). The aneurysm neck was 2 cm. The aorta under the renal arteries and the initial sections of the common iliac arteries were mobilized. The aorta was pinched immediately under the renal arteries and 1 cm above the bifurcation. Thrombectomy was performed. Four lumbar arteries were stitched. The proximal anastomosis between the aorta and the synthetic linear prosthesis of 18 × 9 × 9 mm in the end-to-end type was formed by a continuous winding suture with the “Prolene” 5-0 thread. The prosthesis was clamped, and the aortic clamp was removed. The end-to-end distal anastomosis of the prosthesis and aorta branch was formed by continuous winding suture with the “Prolene” 6-0 thread (Fig. 3C). Before the start of blood flow, heparin 5000 units is injected intravenously to prevent thrombosis.

**Fig. 4:**
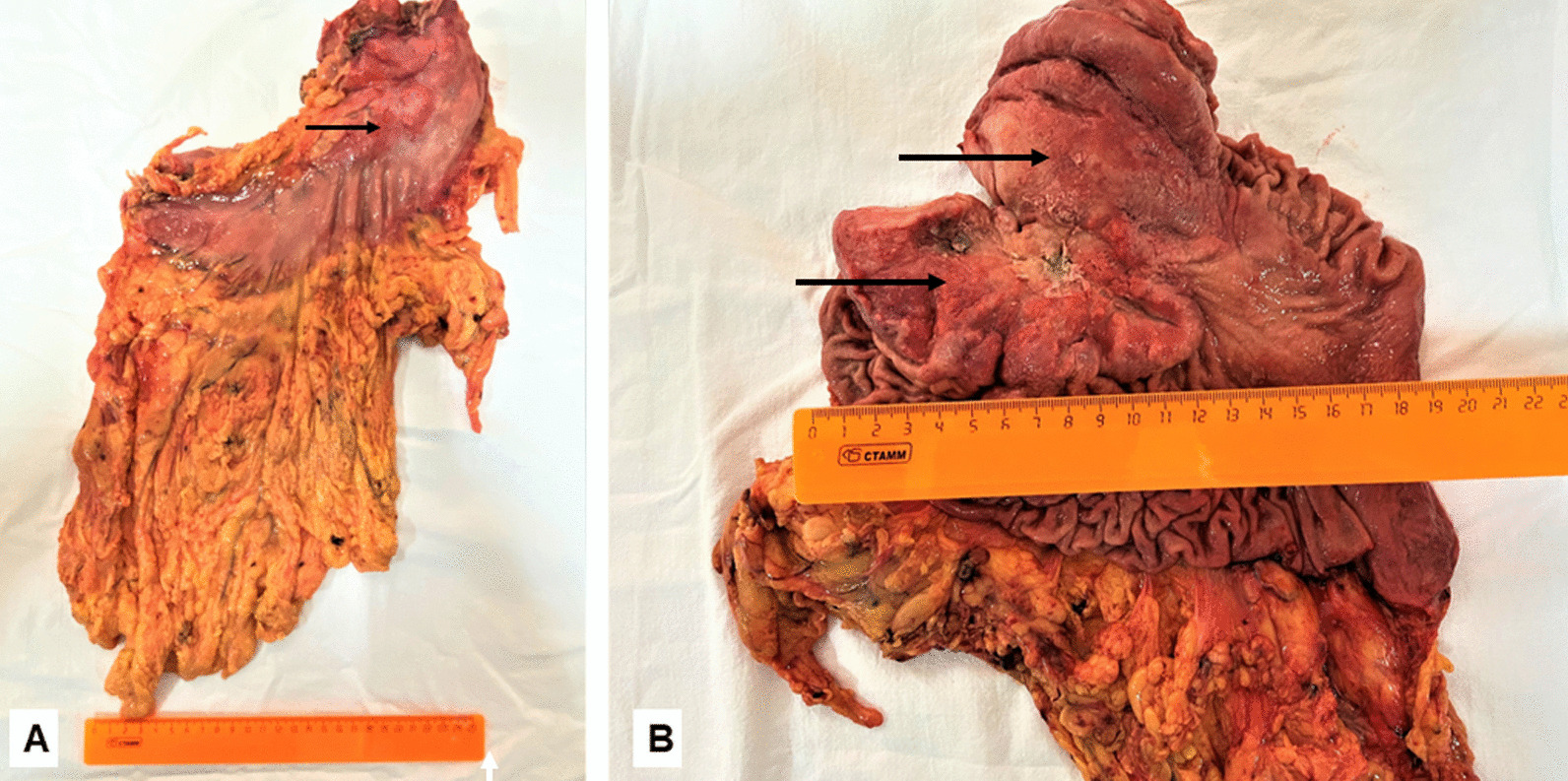
Macroscopically—stomach with tumor (the tumor is indicated by arrows): **A** stomach with greater and lesser omentum; **B** the stomach is cut lengthwise

Histological results: the detected morphological signs corresponded to a low-grade mixed adenocarcinoma of the stomach according to Lauren’s classification, with invasion of subserous tissue (Fig. 4). In 8 of 29 distant lymph nodes, cancer metastases were present (pT3N3aM0 R0 stage IIIB TNM 8).

The postoperative period proceeded without features and complications. After the control x-ray on the 5th day after the surgery, the patient began to eat through the mouth in full. On the 7th day after the surgery, the patient was discharged home in satisfactory condition. The patient received 8 courses of chemotherapy (FOLFOX) and there were no local or distant recurrence by CT of the abdomen and upper gastrointestinal tract endoscopy during 1 year follow up.

## Discussion

Approximately 9–13% of patients with AAA are diagnosed with malignant tumors of abdominal cavity, and the number of patients with a combination of these diseases has increased over the past decades [3–5]. In 1967, for the first time, Szylagyi et al. published a series of observations of patients with aneurysm of the abdominal aorta and malignant tumors of the abdominal cavity [6]. A year later, in 1968, for the first time, Sigler et al. performed a successful simultaneous operation on a 73-year-old patient with aneurysm of the abdominal aorta and intraoperative cancer of the gastric antrum [7]. At the first stage, the resection of the aneurysm with bifurcation prosthetics of the infrarenal aorta was performed, followed by distal subtotal resection of the stomach at the second stage.

Since the second half of the twentieth century, no more than 30 observations of simultaneous operations for abdominal aortic aneurysm and gastric cancer have been described in the world literature [8]. Over the past 10 years, we have only managed to find a few descriptions of the simultaneous performance of gastrectomy for GC and aortic prosthetics for aneurysm, while in the vast majority of cases, the placement of an aortic stent graft has been carried out endovascularly [9, 10].

Despite the fact that both diseases have a very high risk of developing fatal complications, such as bleeding or rupture of an aortic aneurysm, there is no single surgical tactic in the treatment of these patients [3].

According to Perko *et al.*, cumulative 5-year survival of patients with AAA makes up no more than 15%, while the most common cause of death is aneurysm rupture. Based on this, patients with AAA with a diameter of more than 6 cm are recommended surgical treatment in the shortest time from the moment of its detection [11]. The question of simultaneous operation on the aorta and on the stomach, as well as the choice of endovascular or open vascular prosthetics remains open. A number of authors tend to the stage or simultaneous treatment with endoluminal aortic prosthetics, especially in patients with severe concomitant diseases, explaining this as a minimal risk of infection of the prosthesis [9]. However, there are strong arguments in favor of simultaneous traditional interventions. Swanson et al. reported 10 asymptomatic AAA, the rupture of which occurred within 36 days after the primary laparotomy, and according to these authors, was the result of collagen lysis induced by laparotomy, nutritional deficiency and local inflammation, which could have weakened the aortic wall [12, 13]. When choosing a surgical access, there is an opinion that it is advisable to perform gastrectomy through midline laparotomy, while aortic prosthetics is recommended to perform using retroperitoneal access, in order to reduce the risk of aortic graft contamination [14].

From our point of view, the simultaneous intervention for AAA and gastric cancer is justified for a number of reasons. The use of the midline laparotomy provides an adequate and ergonomic workspace for revision, both during the surgery on the stomach and the aorta [15]. In compliance with generally accepted rules of aseptic and antiseptics, the risk of infection of the vascular prosthesis is minimized. From our point of view, endovascular prosthetics is justified only in severe somatic patients. When performing simultaneous intervention (i.e. traditional on the stomach and intravascular on the aorta), requiring additional x-ray equipment in the operating room, the duration of intraoperative anesthesia increases due to the endovascular stage, which can lead to a number of respiratory and neurological complications in the early postoperative period. Taking into consideration the economic component, traditional vascular prostheses are much cheaper than their endovascular analogues, with similar efficiency. Thus, their use is more economically advantageous in case of simultaneous operations.

In case of simultaneous surgery, the question arises: which stage to perform first: vascular or oncological? Our Japanese colleagues believe that it is necessary to perform aortic prosthetics first, which is a clean stage, and after suturing the parietal peritoneum, go to surgery on the stomach [16]. In our observation, the operation began with gastrectomy, since the removal of the stomach as a single unit with a large omentum allowed to free up a significant space in the abdominal cavity, thereby providing convenient access to the aneurysm of the infrarenal aorta and greater freedom of manipulation. After the completion, the abdominal cavity was lavaged with antiseptic solutions, which was a prevention of bacterial contamination of the aortic graft and allowed safe operation on the aorta.

Currently, combined treatment is used for locally advanced forms of stomach cancer, including the surgical stage and a course of chemotherapy. Aortic aneurysm with thrombosis, as well as peripheral vascular thrombosis with aortic aneurysm in some cases, are a contraindication to chemotherapy for cancer patients. Performing a simultaneous operation allowed the patient to undergo rehabilitation after the treatment of two diseases during one hospitalization and, in the shortest possible time, to proceed to the next stage of gastric cancer treatment—chemotherapy, thereby improving the prognosis of life expectancy.

## Conclusion

Thus, traditional simultaneous operations for gastric cancer and aortic aneurysms are technically feasible. Moreover, they have several advantages over stage treatment, such as rehabilitation for two diseases during one hospitalization, economic efficiency due to the lower cost of a traditional aortic prosthesis and the possibility of early chemotherapy for gastric oncology. However, these operations should be carried out in highly specialized medical centers, where surgeons have sufficient experience in both oncological and cardiovascular surgery.

## Data Availability

Data sharing is not applicable to this article as no datasets were generated or analyzed during the current study.

## Abbreviations

AAA: Aneurysm of the abdominal aorta
ASA: American Society of Anesthesiologists Physical Status Classification System
CT: Computed tomography
GC: Gastric cancer
